# Unravelling cardiovascular disease using four dimensional flow cardiovascular magnetic resonance

**DOI:** 10.1007/s10554-016-1031-9

**Published:** 2016-11-25

**Authors:** Vivian P. Kamphuis, Jos J. M. Westenberg, Roel L. F. van der Palen, Nico A. Blom, Albert de Roos, Rob van der Geest, Mohammed S. M. Elbaz, Arno A. W. Roest

**Affiliations:** 1 Division of Pediatric Cardiology, Department of Pediatrics, Leiden University Medical Center, Leiden, The Netherlands; 2 Netherlands Heart Institute, Utrecht, The Netherlands; 3 Department of Radiology, Leiden University Medical Center, Leiden, The Netherlands

**Keywords:** 4-Dimensional flow, Cardiovascular magnetic resonance, Blood flow, Velocity

## Abstract

Knowledge of normal and abnormal flow patterns in the human cardiovascular system increases our understanding of normal physiology and may help unravel the complex pathophysiological mechanisms leading to cardiovascular disease. Four-dimensional (4D) flow cardiovascular magnetic resonance (CMR) has emerged as a suitable technique that enables visualization of in vivo blood flow patterns and quantification of parameters that could potentially be of prognostic value in the disease process. In this review, current image processing tools that are used for comprehensive visualization and quantification of blood flow and energy distribution in the heart and great vessels will be discussed. Also, imaging biomarkers extracted from 4D flow CMR will be reviewed that have been shown to distinguish between normal and abnormal flow patterns. Furthermore, current applications of 4D flow CMR in the heart and great vessels will be discussed, showing its potential as an additional diagnostic modality which could aid in disease management and timing of surgical intervention.

## Introduction

In normal cardiovascular physiology, blood flow in the heart and great vessels shows complex and dynamic three-dimensional (3D) flow patterns, leading to efficient ejection of the blood into the pulmonary and systemic circulation [1]. Congenital or acquired heart disease causes alterations in these blood flow patterns resulting in increased energy loss [2] and reduction of the efficiency of the heart pump by over 10% [1]. Moreover, altered blood flow patterns induce changes to the endothelium, which may increase the risk for cardiovascular incidents later in life [3, 4]. Knowledge of these flow patterns increases our understanding of normal physiology and may help unravel the complex pathophysiological mechanisms leading to cardiovascular disease [1, 5]. However, these complex 3D flow patterns remain challenging to visualize and characterize. Four-dimensional (4D) flow cardiovascular magnetic resonance (CMR) has emerged as a suitable technique for comprehensive visualization and quantification of blood flow and energy distribution in the heart and great vessels in healthy subjects as well as in patients with cardiovascular disease [6].

The influence of altered aortic flow patterns on pathophysiology has been investigated most intensely in patients with a bicuspid aortic valve (BAV) [3, 7, 8] and in patients with Marfan syndrome [9–13]. For example, in patients with Marfan syndrome, flow parameters have been linked to an increased aortic size, as shown by 4D flow CMR [9–13]. Because of the complexity of the heart’s atria and ventricles, assessment of intra-cardiac blood flow characteristics is more challenging, but knowledge of such blood flow is of utmost importance in diseases like ischemic heart disease, dilated cardiomyopathy, congenital heart defects (CHD) and pulmonary hypertension.

In this review, the challenges in the application of 4D flow CMR to study hemodynamics in the cardiovascular system are discussed, as well as the visualization and quantification methods. Furthermore, current insights in normal flow patterns, flow disturbances due to cardiovascular disease and its consequences, as assessed with 4D flow CMR, will be addressed.

## State of the art

Four-dimensional flow CMR, phase-contrast (PC) CMR with velocity-encoding in all three spatial directions, resolved relative to all dimensions of space and the dimension of time along the cardiac cycle, represents all directions and spatial regions of flow within the boundaries of the defined volume [14, 15].

### Acquisition parameters

Recently, a consensus statement was published, stating the clinical and scientific significance of 4D flow CMR and providing recommendations for its use [15]. In this consensus statement, a list of acquisition parameter settings as a baseline 4D flow CMR protocol is proposed against which alternative protocols can be compared. Optimized parameter choices are recommended for special populations (e.g. children) or analysis of advanced flow parameters [15]. An important parameter is the Venc, which represents the maximum flow velocity that can be acquired without having to correct for phase wrapping. If the Venc is set too low, velocity aliasing will occur, however when the Venc is set too high, the level of velocity noise will increase [14]. A Venc that is set 10% higher than the maximal expected velocity is recommended [15].

Evaluation of flow patterns in the cardiac chambers requires a spatial resolution of <3.0 × 3.0 × 3.0 mm^3^ and <2.5 × 2.5 × 2.5 mm^3^ for the aorta or pulmonary artery. In order to extract feature information (i.e., stroke volume, peak velocity, peak flow rate, etc.) of flow (velocity) curves from the velocity field, a high temporal resolution is required. This resolution is defined by the repetition time, the number of velocity encodings and, in case of segmented acquisition, the number of segments. Since 4D flow CMR is to be applied to these large anatomical regions in the human body with adequate spatial and temporal resolution and potentially with some form of respiratory motion compensation, the acquisition time required to collect all this flow information is typically long (i.e., 10–25 min) and may be too demanding on patients or on the clinical workflow.

Three-directional encoding without any acceleration technique would be the most accurate approach, with the best signal-to-noise ratio (SNR) and the least amount of phase offset errors [15]. However, this is generally not feasible in a clinical setting. Therefore, to make 4D flow CMR applicable for clinical use, several methods are available to reduce acquisition time. Such accelerating techniques include parallel imaging using multi-element phased array coils (SENSitivity Encoding, SENSE) [16] or k-t undersampling methods like k-t BLAST (Broad-use Linear Acquisition Speed-up Technique) [17]. Acquiring read-outs of multiple k-lines per RF excitation may accelerate the acquisition as well, however, at the penalty of reducing temporal resolution and/or signal-to-noise. Furthermore, different acquisition strategies apart from the standard Cartesian k-space read-out, like echo planar imaging (EPI) [18], spiral [19] or radial [20] (e.g., PC-VIPR, vastly undersampled isotropic projection reconstruction) read-out methods can further reduce acquisition time.

Another way to accelerate the acquisition is to acquire free-breathing 4D flow CMR with sophisticated respiratory gating or even without any respiratory motion control. Compensation of respiratory motion, which is used to reduce motion artifacts and improve accuracy, is usually difficult to achieve without significantly increasing scan duration. The most commonly used method for this motion suppression is respiratory gating by a navigator, however this increases acquisition duration substantially. Respiratory self-gating methods allow sampling of 4D flow data over the entire cardiac cycle, usually using center K_0_ point, center K_0_ profiles or low-resolution images to derive the breathing motion and then to adjust the acquisition scheme in real-time to reacquire motion-corrupted data, allowing free breathing while acquiring 4D flow data within clinically acceptable acquisition time [21, 22]. However, recently it was shown that 4D flow CMR without any respiratory gating may be performed while preserving accurate quantitative results from stroke volume assessment in the great vessels [23] and in whole-heart 4D flow [24].

### Sources of error

Several sources of error can affect the 4D flow data and should be corrected for. Major sources of error are: eddy current effects, concomitant gradient field effects, gradient field non-linearity and phase wraps [15]. Inhomogeneities in the magnetic field and eddy current effects in the receive coil will result in background phase distortion [25]. Concomitant gradient fields are a result of Maxwell’s equations for the divergence and curl of the magnetic field and lead to background offsets [26]. Furthermore, a non-linear gradient field can induce deviations from the nominal gradient strength and orientation causing deviations in velocity quantification [27]. Some of these errors are partially corrected by reconstruction algorithms implemented on the Magnetic Resonance Imaging (MRI) scanner software. Background phase offset errors are usually corrected by either performing a phantom velocity-encoded scan simulating static tissue and using this data set as a reference for background subtraction, or by the approach of fitting a multi-order polynomial through areas identified as static tissue, for correcting the local phase signal [28].

Finally, velocity aliasing, or phase-wrapping, will occur when blood flow velocities exceed the a priori set Venc value. The use of a phase-unwrapping algorithm is recommended prior to image analysis. Identification of abrupt phase shifts in the temporal and/or spatial domain is a common way to identify areas with phase wrapping [29]. Aliasing correction should be performed in the original source images of each individual encoding direction.

## Visualization and quantification

Several tools are developed to help visualize velocity vector fields of blood flow in the heart and vessels which makes qualitative assessment of flow patterns possible. Visualization is needed in order to characterize blood flow parameters.

**Fig. 1:**
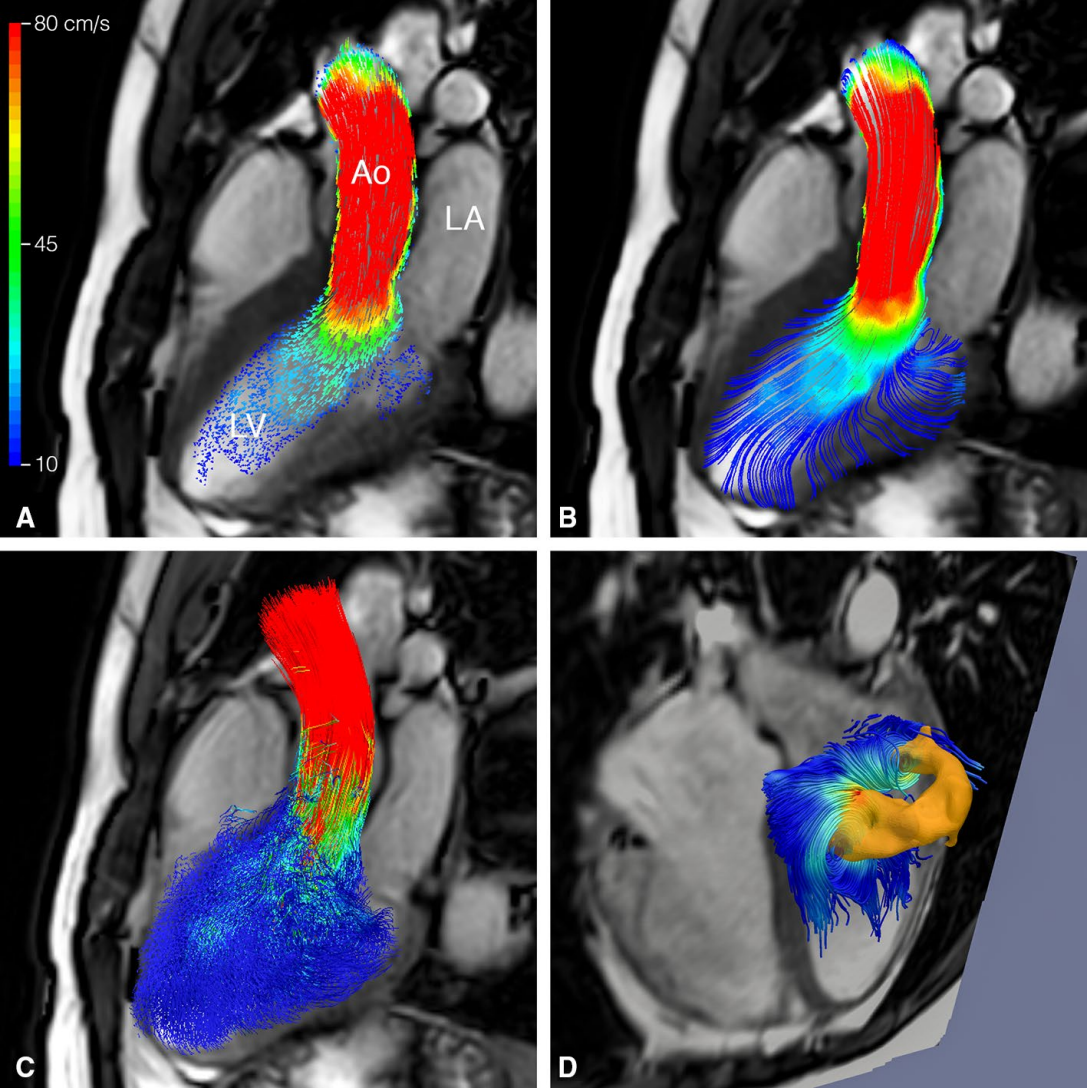
Visualization of left ventricular systolic blood flow using 4D flow CMR in a healthy volunteer (24-year-old man). In **a** blood flow velocity is displayed by *color-coded* vector glyph representation. Direction and velocity magnitude are presented by vector *size* and *color*. In **b** a streamline representation is presented and **c** shows a pathline representation. Isosurfaces can be used to display flow structures with a common property, such as vorticity. In **d** a *ring-shape* vortex is displayed in late diastolic LV filling in the same volunteer. Vortex extraction is performed using Eulerian vortex core analysis. The vortex ring is displayed with streamlines superimposed. *LA* left atrium, *LV* left ventricle, *Ao* aorta

Most common visualization types are the vector glyph representation, or the use of streamlines or pathlines (Fig. 1). A vector glyph represents the magnitude and direction of the velocity measured from each voxel. However, a cine representation of vector data may be difficult to interpret, as data may quickly become cluttered.

**Fig. 2:**
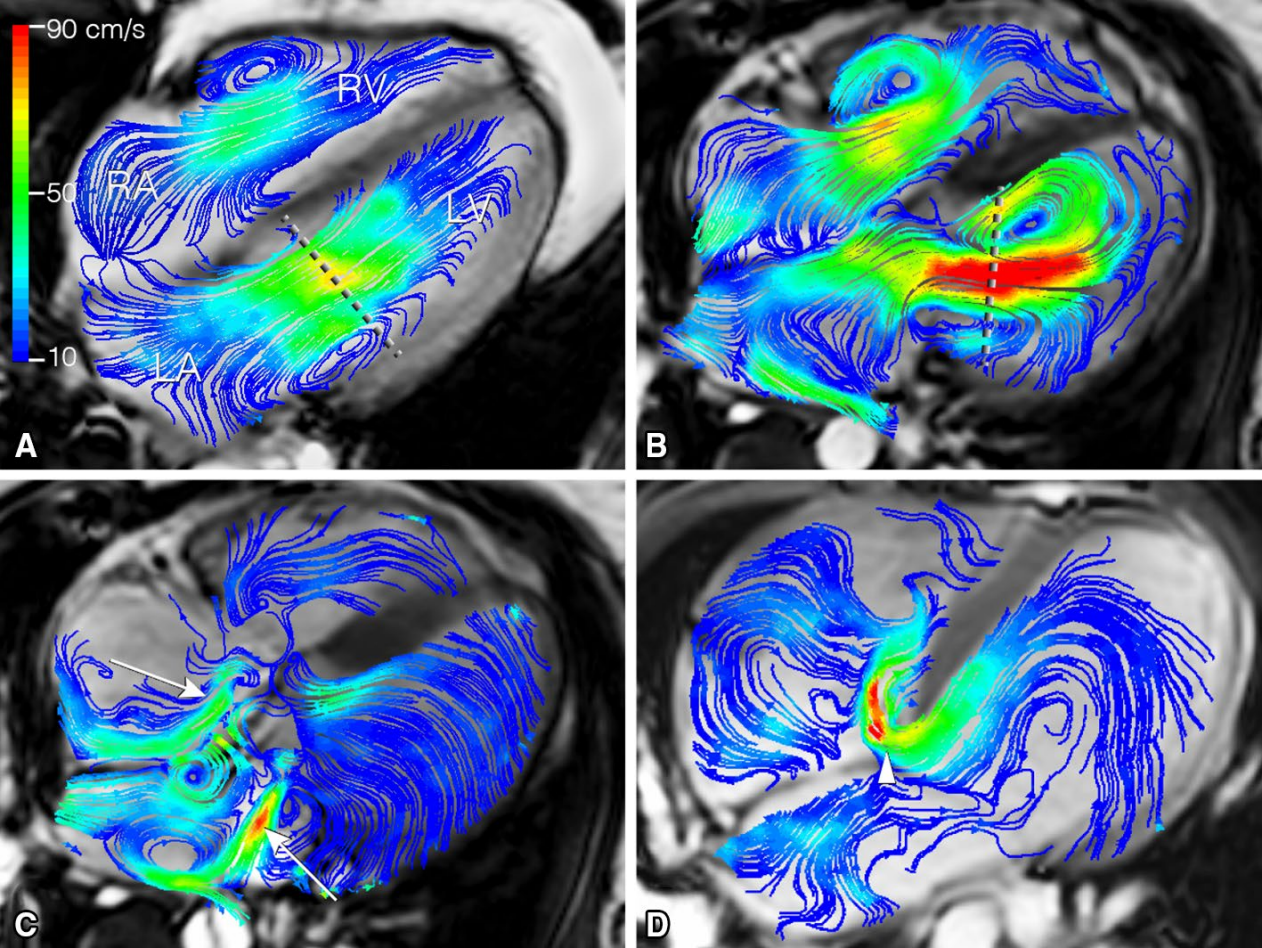
Left ventricular (LV) inflow assessment from 4D flow CMR and retrospective valve tracking. Streamline visualization with *color coding* of early LV inflow shows central inflow in a healthy volunteer (in **a** 53-year-old man) and a laterally directed inflow in a patient with corrected atrioventricular septal (AVSD) defect (in **b** 26-year-old woman). Positioning of the reformat plane is displayed by the *dashed line*. In **c** eccentric regurgitant jets in both atrioventricular valves during systole in the same corrected AVSD patient are displayed (*arrows*). In **d** shunt flow in an uncorrected AVSD is displayed (*arrowhead*) in a 24-year-old male patient. *LA* left atrium, *LV* left ventricle, *RA* right atrium, *RV* right ventricle

Streamlines are curves which are tangent to the velocity direction at a particular point in time, representing the blood flow direction at an instant of time [30, 31]. Streamline visualization can be used for visualization of inflow and outflow direction, regurgitant jets and circulating flow patterns at specific time points in the cardiac cycle [32, 33]. In the aorta, streamline visualization is often used to show helical flow patterns [34]. Streamline visualization in combination with retrospective valve tracking allows for accurate quantification of net flow volumes through each of the four heart valves (Fig. 2) [35, 36]. Retrospective valve tracking is a method in which the scanned 3D volume is retrospectively reformatted into two-dimensional (2D) measurement planes with through-plane velocity encoding to allow for trans-valvular flow quantification [35, 36]. Measurement planes can be adjusted per individual phase, following the valve position, inflow direction and the dynamically changing regurgitant jets. Optimized positioning of these planes should be based on the direction of the peak velocity visualized by streamlines [37].

Different from streamlines, pathlines show the path a particle (i.e., a voxel) has followed over time [30]. Particle paths or pathlines are generated by backward/forward particle tracing using integration methods to calculate displacement from the velocity data [30]. For intra-cardiac blood flow, typically, at end diastole, each voxel inside the LV is considered to represent a seed point (i.e., a particle). Pathlines are then calculated by integration over time: backward tracing over the diastole and forward tracing over systole. Pathlines are also frequently used to evaluate complex flow patterns, such as helical flow patterns in the aorta and pulmonary artery [34, 38].

**Fig. 3:**
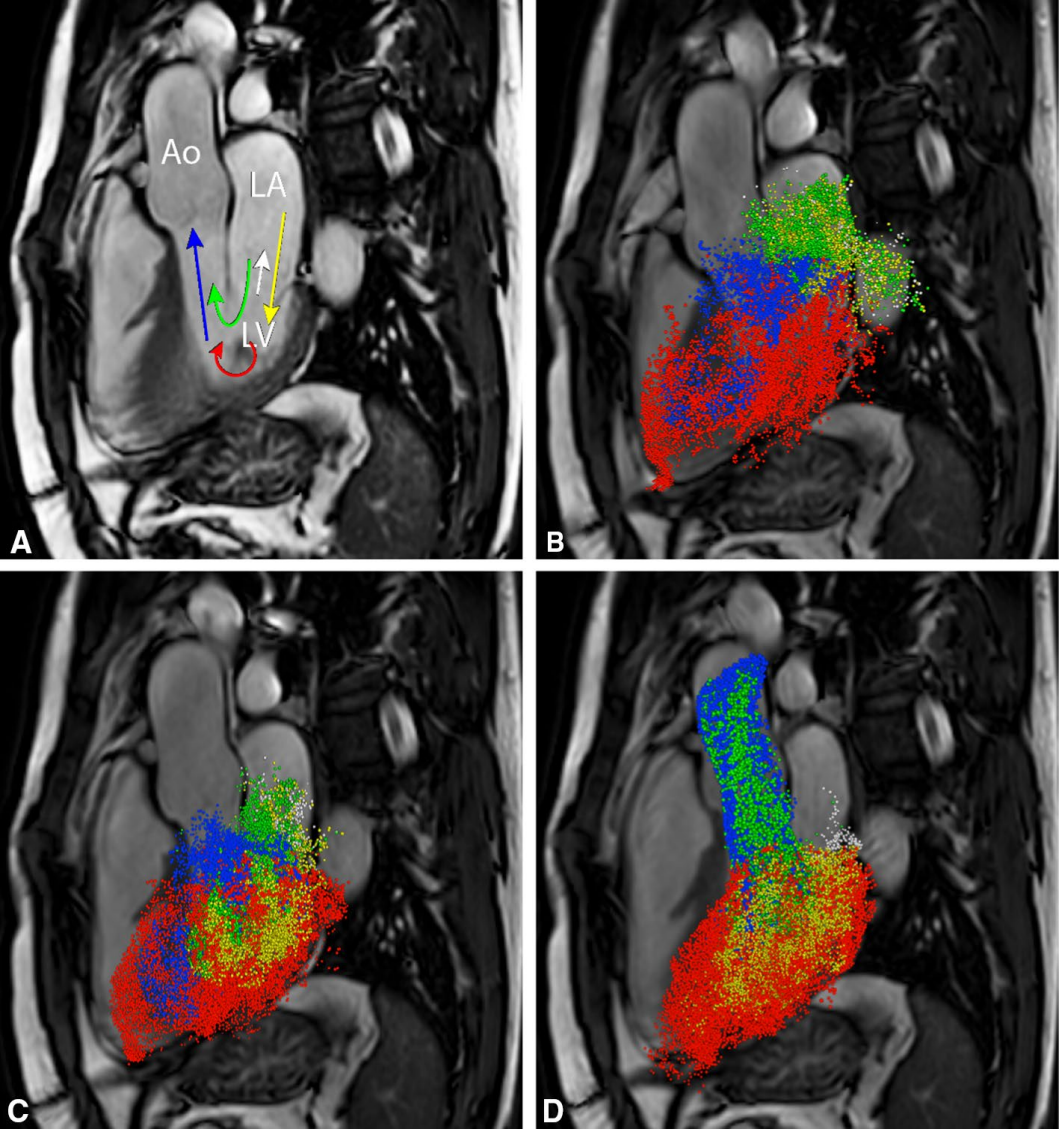
Multi-component particle tracing of left ventricular (LV) blood flow in a healthy volunteer (26-year-old man). In **a** five components of LV are schematically presented. *Green* direct flow, *Yellow* retained inflow, *Blue* delayed ejection flow, *Red* residual volume, *White* regurgitation. Three time-points in cardiac cycle are represented: diastole in **b** end diastole in **c** systole in **d**. *LA* left atrium, *LV* left ventricle, *Ao* aorta

Another unique feature of particle tracing in intra-cardiac blood flow is the possibility to discriminate different parts of blood flow with some specific functional property based on where the seed points are flowing towards and where they came from. Different components in the blood flow organization in the left ventricle (LV) [39] and the right ventricle (RV) [40] can be discriminated, such as the 4-component evaluation (Fig. 3) as introduced by Bolger et al. [41] for the LV:

1. *Direct flow* blood that enters the LV through the mitral valve during diastole and is ejected from the LV into the aorta during the subsequent systole in the analysed heartbeat;
2. *Retained inflow* blood that enters the LV during diastole but is not ejected during the subsequent systole in the analysed heartbeat;
3. *Delayed ejection flow* blood that starts and remains inside the LV during diastole but is ejected during the subsequent systole;
4. *Residual volume* blood that remains within the LV for at least two subsequent cardiac cycles.

A fifth component can be added, *Regurgitation* blood that leaves the LV through the mitral valve into the atrium during systole [42]. It should be taken into account that particle tracing analysis requires high temporal resolution and adequate signal-to-noise, as results coming from an integration procedure on noisy data and over large time steps may not be reliable.

From the 3D flow velocity field, helical and vortical flow patterns can be identified in normal and pathological blood flow. An important intra-cardiac flow pattern is vortex flow: a group of fluid particles swirling around a common axis. Two methods have been used to analyze and visualize intra-cardiac vortex flow patterns: Lagrangian and Eulerian. Lagrangian coherent structures (LCS) can be used to quantify and visualize the total amount of flow that entrains into a vortex ring flow structure over a period of time [43]. Eulerian vortex core analysis allows quantitative characterization of instantaneous 3D vortical flow patterns and its intra-cardiac evolution over time [44]. Altered intra-cardiac 3D vortex flow properties have been identified in the presence of abnormal valvular morphology and were associated with adverse blood flow efficiency [2, 44, 45].

Four-dimensional flow CMR is also used for studying energetics in the blood flow. The kinetic energy (KE) of a moving particle with a certain mass *m* (particle volume multiplied by the blood density) and velocity *v*, can be calculated with the formula ½*mv* ^2^. The KE at a specific time point can then be calculated by summing the KE of each voxel within a specified anatomical region. Viscous energy loss (EL) is the kinetic energy that is lost due to frictional forces among blood particles and surrounding structures in the ventricle, induced by the blood viscosity. EL can be calculated from the Navier–Stokes energy equations [2]. Turbulent kinetic energy (TKE) is another frequently used energy parameter used to quantify the energy lost due to turbulent flow and is calculated from dedicated reconstructions of the intravoxel distribution of spin velocities [46].

Wall shear stress (WSS) is a quantitative value for the shear forces of the blood flow acting on the vessel wall [47]. It can be used to quantify the impact of flow on the vessel wall and it has been shown to correlate with changes in the extracellular matrix (ECM) and endothelial cells [3]. Higher blood flow velocity will increase WSS [48].

Aortic wall elasticity, an important mechanical property of the vascular wall, can be measured with traditional 2D one-directional velocity-encoded CMR, [49] but also by multi-directional velocity-encoding or 4D flow CMR, [50] by measuring the propagation speed of the systolic wave front along the course of the aorta. This biomarker for arterial stiffness is called the pulse wave velocity (PWV) [51]. A shorter propagation time, thus higher PWV, is indicative of a stiffer aorta and presence of atherosclerosis [52].

## Applications

In the following section normal and abnormal blood flow characteristics as assessed with 4D flow CMR will be reviewed. We will describe the use of 4D flow CMR in assessing normal intra-cardiac and intravascular flow patterns, as well as applications in acquired and congenital cardiovascular disease.

### Atrial flow patterns

In the normal human heart, blood flow in the left atrium (LA) follows specific paths from the pulmonary veins to the mitral valve. The occurrence of atrial vortices has been shown, which may be beneficial in avoiding atrial stasis [53]. In the LA, inflow from the right pulmonary veins follows the atrial wall from its inlet near the inter-atrial septum toward the mitral annulus, while inflow from the left pulmonary veins suddenly shifts towards the mitral valve after entry through the lateral left atrial wall, as was shown with particle tracing analysis [53]. In the right atrium (RA), blood flow from the inferior vena cava (IVC) and superior vena cava (SVC) turns anterior after entering the atrium, which causes a forward rotating movement of the anterior part of the right atrial blood volume towards the inlet of the tricuspid valve [54].

Assessment of atrial flow patterns and blood flow velocity is important in patients with atrial fibrillation (AF), since AF is associated with an increased risk of embolic stroke due to thrombus formation in the LA [55]. Patients with AF show global and regional changes in atrial flow dynamics, such as decreased blood flow velocities and increased stasis, which can be evaluated with 4D flow CMR and could be a helpful indicator in risk assessment for thrombogenesis in these patients [56–58].

In patients with mitral regurgitation, severely disturbed flow patterns in the LA with elevated values of TKE develop, related to the severity of regurgitation [59]. These atrial flow effects of mitral regurgitation assessed by 4D flow CMR could potentially be used in risk assessment for the onset of decompensated heart failure in patients with prior asymptomatic mitral regurgitation [59]. The amount and severity of mitral valve regurgitation (i.e., the regurgitant flow volume and the regurgitant flow fraction) can accurately be assessed with the use of 4D flow CMR with retrospective valve tracking [35, 36].

In corrected atrioventricular septal defect (AVSD) patients, regurgitation of the left atrioventricular valve (LAVV) is common [60]. In these patients, the regurgitant jets are dynamic and eccentric (Fig. 2) and have a non-circular cross-sectional shape, which makes them challenging to quantify with echocardiography [61]. However, the regurgitant fraction and the volume of the complex regurgitant jets can be quantified accurately with the use of 4D flow CMR with retrospective valve tracking [33]. Furthermore, 4D flow CMR can also be applied to investigate intra-cardiac baffle constructions for leakage and obstruction, for instance after double switch operation for congenitally corrected transposition of the great arteries [62].

### Flow patterns in the left ventricle

The complex geometry of the normal left ventricle (LV) causes asymmetric blood flow, which promotes efficient ejection of blood in the systemic circulation and minimizes the energy dissipation [1, 54]. In the normal LV, 30–35% of the LV end diastolic volume represents blood flow that enters the LV during diastole and is ejected into the aorta during systole in the subsequent heartbeat (i.e. direct flow) [39, 42]. Using 4D flow CMR, vortical flow patterns have been described that form during diastole, with a close relation to the motion of the anterior mitral leaflet and the shape of mitral inflow [43, 44, 63]. During diastole, a pair of counter rotating vortices has been consistently reported to form distal to the mitral valve. In three-dimensions, this pair of vortices extend to form a ring-like vortex shape. 4D Flow CMR has enabled the characterization of the instantaneous time-evolution of 3D vortex ring flow within the LV over the complete diastole [44]. Formation of vortex ring flow has been suggested to help efficient MV closure and diastolic filling, minimize kinetic energy loss and prevent thrombus formation [1, 54, 64, 65]. Vortex flow patterns can change due to age, gender, blood pressure, ventricular geometry and mitral/atrioventricular valve abnormalities [45, 66].

In patients after AVSD correction, the LV inflow over the trans-left atrioventricular valve (LAVV) is altered (i.e., a more lateral inflow was shown by streamline visualization) (Fig. 2) [32]. 4D flow CMR with particle tracing showed that this altered inflow after AVSD correction also affected the intra-cardiac flow organization, which presented as reduced direct flow and increased retained inflow in the apical and lateral region of the LV cavity [42]. Despite that global cardiac function parameters (including ejection fraction, end diastolic volume, stroke volume and cardiac output) were within the normal range in these patients, significantly altered vortex ring flow properties were found and associated with a 2–4 fold increase in viscous energy loss levels compared to healthy volunteers [2, 45]. This might indicate that properties of vortex ring flow within LV blood flow could be a subclinical marker of cardiac (dys)function preceding decline in global functional parameters [2].

In patients with systolic or diastolic dysfunction, flow disturbances can be evaluated with 4D flow CMR imaging. LV diastolic dysfunction in patients with normal systolic LV function is a risk factor for mortality [67]. LV diastolic function parameters, such as early (E) and late (A) filling rates, E/A ratio, and E-peak acceleration and deceleration duration, can be assessed accurately with the use of 4D flow CMR with retrospective valve tracking [68]. Furthermore, a study using 4D flow CMR with color vector visualization showed that in patients with various stages of diastolic dysfunction, LV diastolic flow only extends a short distance in the LV and stops in the middle of the LV cavity due to decreased flow acceleration [69]. Patients with dilated cardiomyopathy showed a smaller direct flow volume and greater end-diastolic KE distribution in the residual volume, despite normal LV stroke volume, as shown by particle tracing analysis with the 4-component model [70]. Whereas, in patients with ischemic dilated cardiomyopathy, altered flow patterns were related to complex and asymmetric vortex rings and decreased vortex volume [43].

Patients who have had a Fontan operation, a palliative treatment for patients with single-ventricle physiology, have complex and heterogeneous underlying ventricular morphologies which makes studying the intra-cardiac blood flow in these patients challenging. However, the dynamic and 3D nature of the blood flow in these patients makes 4D flow CMR particularly suitable for the assessment and quantification of these flow patterns. Recently, various blood flow patterns were shown in these patients with 4D flow CMR with streamline visualization and inflow volumes were quantified with retrospective valve tracking [37]. Assessment of the caval blood flow in these complex patients will be addressed in the section on intravascular blood flow patterns.

### Flow patterns in the right ventricle

Visualization of flow in the right ventricle (RV) remains challenging because of the complex 3D shape of this ventricular cavity. In vivo and in vitro studies showed that in the normal RV, blood flow rearranges along the converging outflow tract during systole to form helical circulating flow towards the pulmonary orifice [5, 40, 54]. Particle tracing analysis in the RV showed that 44% of the blood is direct flow, which moves from the RA into the RV during diastole and moves towards the RV outflow tract, rounding the infundibular septum and contributing to vortical formation that extends in the outflow tract [40]. Compared to the other flow components, this direct flow possesses a larger presystolic KE, which may benefit the efficiency of systolic ejection [40].

Extensive knowledge of the RV flow and function is of interest in many types of heart defects, especially in patients with CHD, as lesions affecting the RV are an independent risk factor for early attrition [71]. For example, patients with Tetralogy of Fallot (ToF) have altered RV flow patterns resulting in increased vortical flow patterns in the RA and RV during diastole [72]. Accurate assessment of forward flow and regurgitation fraction over the tricuspid and pulmonary valve in these ToF patients after corrective surgery, which is important in the assessment of RV diastolic functional impairment, can be performed using 4D flow CMR with retrospective valve tracking [73].

In patients with pulmonary hypertension (PH), RV diastolic dysfunction (RVDD) is an important prognostic factor [74]. Recently, it was shown by 4D flow CMR that patients with RVDD due to PH have altered vorticity in the RV at peak E- and A-diastolic filling. The presence of altered RV vorticity could be a valuable marker to evaluate the risk of RVDD development, as it was shown to have a clear relationship [75]. Furthermore, 4D flow CMR showed that PH is related to altered KE RV work density (i.e., a measure of the amount of work the RV has produce to transport blood from RA to the pulmonary artery) and viscous energy loss in the blood flow in the pulmonary artery,[76] which is also shown to be related to increased vorticity in the blood flow in the pulmonary artery [77].

In patients with ischemic heart disease, 4D flow CMR could be used to detect impairment of RV function, as shown by changes in flow distribution and KE, which could potentially have prognostic implications [78].

### Flow patterns in the great vessels

#### Aorta

Normal aortic flow patterns include right-handed helical outflow and late systolic retrograde flow (blood flowing counter to the main forward stream), as shown by 4D flow CMR [34, 79]. This helical and retrograde flow results from the curvature of the arch, the pulsatility of the blood flow and the compliance of the aortic wall [79]. Aging has been shown to influence flow patterns in several ways; direction of the helical flow may change from right-handed to left-handed, [80, 81] the aortic velocity distribution may change, resulting in changing WSS maps [82–84] and PWV values along the aorta increase [85, 86]. Therefore, age has to be taken into account when evaluating aortic flow patterns in healthy subjects and patients with cardiovascular disease.

The application of 4D flow CMR in patients with aortic disease is promising as it can help gain knowledge of the disease progression, it can aid the prediction of adverse aortic events and can be useful in the optimization of individualized management strategies. Most extensive aortic 4D flow research has been done in patients with BAV [3, 7, 8] and Marfan syndrome [9–13].

**Fig. 4:**
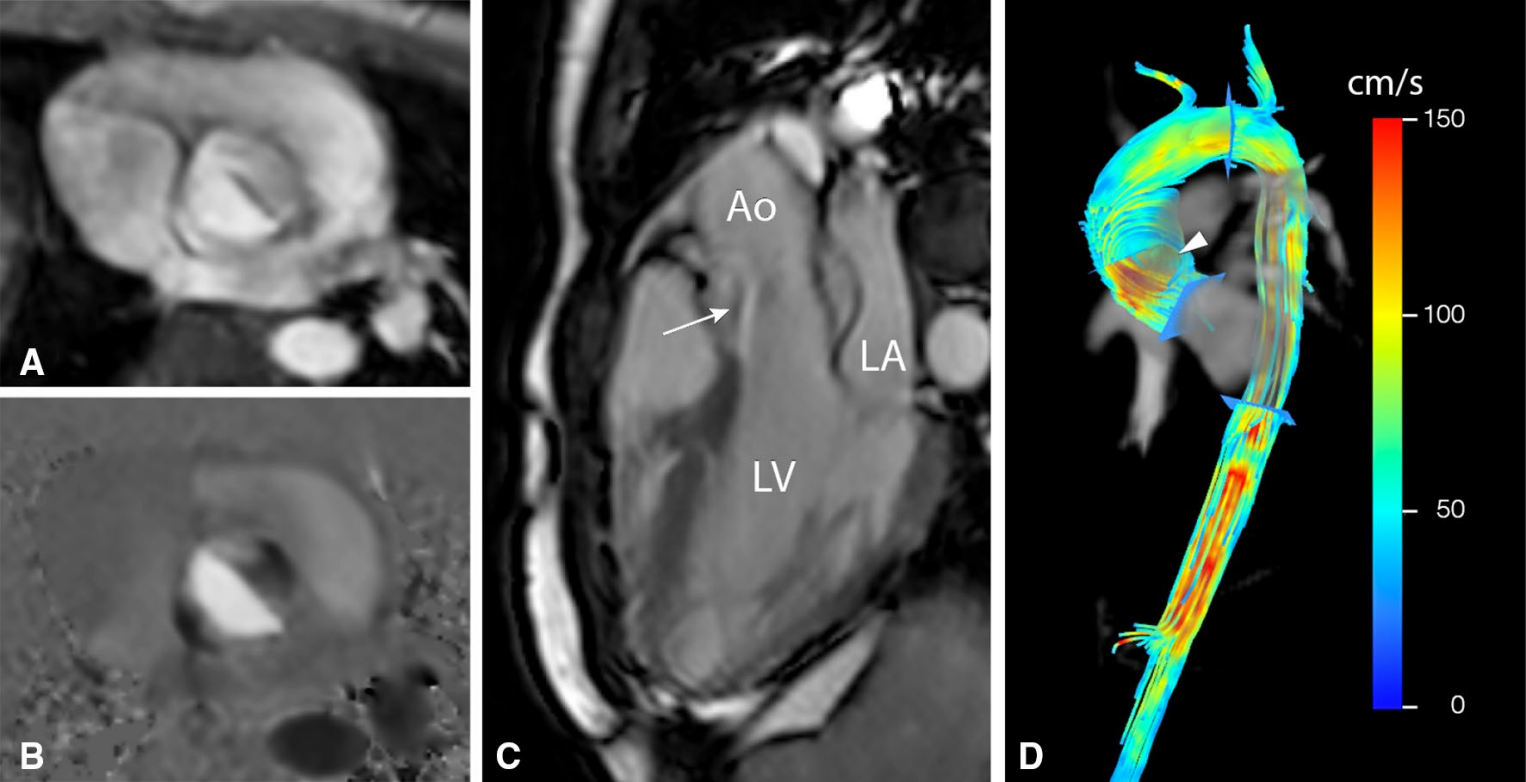
Patient with bicuspid aortic valve (BAV, 28-year-old man) and mild dilatation of the ascending aorta. **a** and **b** show axial views of through-plane velocity-encoded phase contrast MRI acquisition of the flow velocity through the valve (**a** magnitude image, **b** phase image). In **c** a double-oblique lateral view of the aortic outflow tract with BAV is presented. **d** Shows streamline visualization with *color coding* of systolic outflow, with abnormal circulating blood flow (*arrowhead*) and helical flow in the aortic arch

Patients with BAV frequently develop aortic valve dysfunction, ascending aortic aneurysms, and aortic dissection. For many years, aortic dilatation in these patients has been attributed to the genetic susceptibility resulting in a concomitant abnormal development of the ascending aorta and BAV. 4D flow CMR shed another light on this hypothesis by identifying different abnormal outflow patterns in the ascending aorta (Fig. 4) that might predispose to this aortopathy [7, 8]. Recently, it has been shown that different fusion patterns of the aortic valve will lead to different impingement flow jets on the ascending aortic wall [3, 8]. Fusion of right and left leaflets causes right-handed helical flow and right-anterior flow jets, while right and non-coronary leaflet fusion causes left-handed helical flow with left-posterior flow jets [8]. These regions of altered flow patterns show elevated WSS which correlates well with extracellular matrix changes in that aortic region [3]. This suggests a hemodynamic contribution to the aortopathy.

In patients with Marfan syndrome, an inherited connective tissue disease at risk for thoracic aortic dilatation, local helix flow in the ascending aorta as well as abnormal regional WSS has been linked to increased aortic size [9–11]. Furthermore, in young patients with Marfan syndrome, hemodynamic differences in WSS were found at specific regions along the thoracic aorta that correspond to the locations where aortic dissection and aortic rupture often originate in these patients, i.e., the proximal ascending aorta and proximal descending aorta [13]. These data, together with the report of a single Marfan syndrome case in which prior to an aortic dissection type B, formation of abnormal flow patterns and altered WSS in the proximal descending aorta was observed, suggest that hemodynamic factors may play a predictive role in the onset of adverse events [12].

**Fig. 5:**
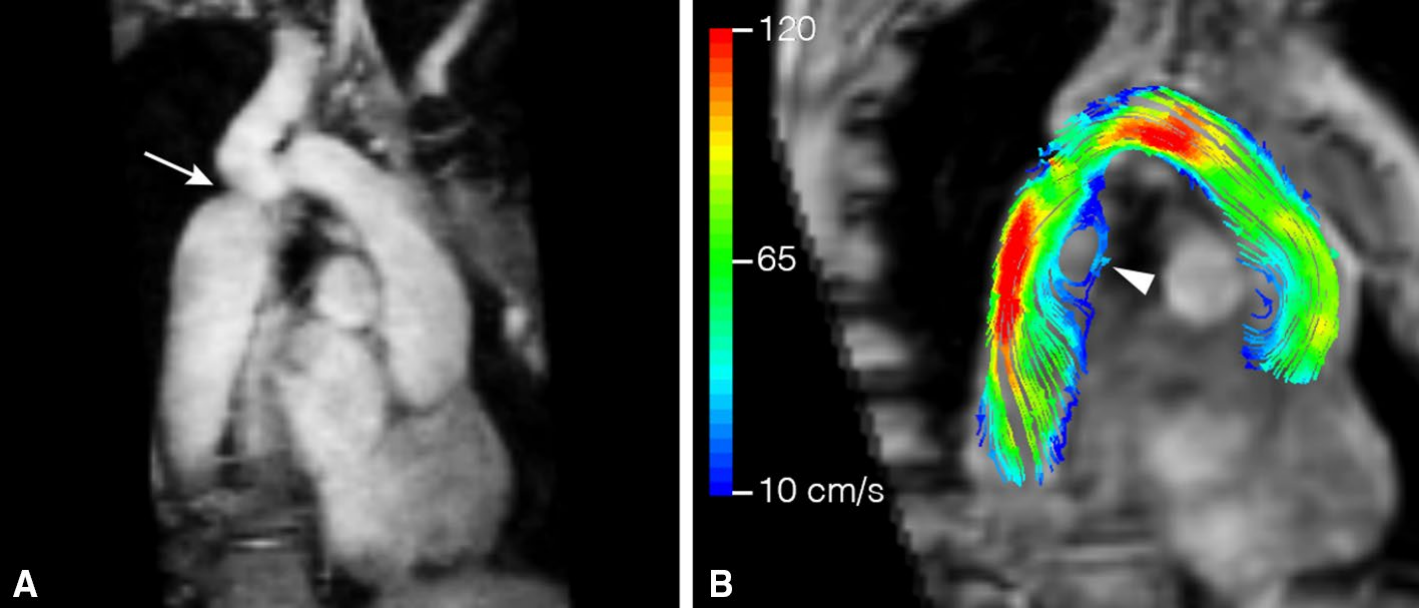
Patient with a coarctation of the aorta (CoA, 29-year-old woman). In **a** a bright blood image of the aortic arch and proximal descending aorta with CoA (*arrow*) is presented, **b** shows the streamline visualization with *color coding*. Distal to the CoA, abnormal flow with recirculation is presented (*arrowhead*)

In patients with coarctation of the aorta (CoA), a short segment of narrowing of the proximal descending aorta just beyond the origin of the arteries that supply the head and arms, 4D flow CMR is an accurate method for the evaluation of collateral flow, which is related to hemodynamic significant coarctation [87]. Also, altered flow patterns and increased WSS can be found in patients with CoA in the entire aorta, before and after repair (Fig. 5) [88]. Other promising applications of aortic 4D flow CMR are related to noninvasive investigation of trans-stenotic pressure gradients in the presence of stenosis in vascular diseases such as aortic CoA [89, 90]. Today, severity of stenosis in CoA is estimated by ultrasonography but the pressure gradients are often overestimated compared to the actual measurements in vivo per catheter. This potentially leads to unnecessary early interventions, with its risk for the need of more complex and increased amount of re-interventions per patient. Thus, predicting the need and timing of intervention for aortic CoA non-invasively can be optimized with 4D flow CMR. Furthermore, energy losses that appears in these turbulent flow conditions can be quantified and maps for dissipation of kinetic energy can be created [91, 92]. These applications make it possible to simulate with advanced post-processing software (by virtual interventions) which intervention preserves the natural thoracic aortic function the most, prior to the intervention [93].

#### Pulmonary artery

In normal pulmonary physiology, two counter-rotating helical flow structures in the main pulmonary artery (PA) were shown with 4D flow CMR, which both contribute mainly to the flow in the right pulmonary artery (RPA) [94]. In early systole, blood flow from the right side of the PA is distributed to the RPA and blood flow from the left side of the PA is distributed to the left pulmonary artery (LPA), while later in systole blood flow from the left posterior side of the PA is distributed to the RPA as shown by streamline visualization [94]. Changes in the pulmonary blood flow are age dependent, which may be helpful in future studies in understanding pathological blood flow in patients with pulmonary disease [95].

Patients with pulmonary hypertension (PH) are currently diagnosed when mean pulmonary artery pressure (mPAP), measured invasively by right heart catheterization, exceeds 25 mmHg [96]. 4D flow CMR with streamline visualization showed abnormal vortical flow in the main PA of these patients. The presence, and in particular the duration of vortical flow presence, could become a useful noninvasive diagnostic marker as it has been shown to correlate well with mPAP [77]. Also, a decrease in vorticity in the main PA and RPA as assessed by 4D flow CMR was recently associated with an increase in pulmonary vascular resistance (PVR) in patients with PH [97].

Altered flow patterns have also been described with 4D flow CMR in the PA of patients with repaired ToF [72]. The increase of these abnormal flow patterns, specifically helical and vortical flow, could be related to the size of the pulmonary arteries or increased PVR and elevated PAP [72].

In patients with a Fontan circulation, blood flows passively from the IVC and SVC to the pulmonary arteries without passing through a ventricle. As expected, this results in altered pulmonary and caval blood flow patterns [38]. As these patients require lifelong follow-up, accurate visualization and quantification of flow patterns is crucial. Several 4D flow CMR studies have shown that blood flow from the SVC favors the right pulmonary artery (RPA), while most of the IVC blood flows to the left pulmonary artery (LPA), as shown by particle tracing analysis [98, 99]. Recently it was shown that the cross-sectional area of the pulmonary arteries in these patients is related to altered flow distribution [99]. The study of caval blood flow distribution could help to identify the patients at risk for Fontan failure or the development of pulmonary arteriovenous malformations, an important complication in these patients leading to systemic oxygen desaturation [100].

## Conclusions and future application/advances

In the recent years, 4D flow CMR has emerged as a suitable technique for research use and several studies have shown its clinical value in patients with congenital and acquired heart disease. Shorter acquisition duration has made application feasible in the clinic. However, dedicated studies investigating the reproducibility and reliability of some of the 4D flow CMR parameters are still warranted before 4D flow CMR can be applied in daily clinical practice. In this review, we showed the different advantages and possibilities of 4D flow CMR, intra-cardiac as well as intravascular. Knowledge of normal and abnormal blood flow has increased the understanding of normal physiology and is necessary for the distinction between cardiovascular health and disease. 4D flow CMR is a promising additional diagnostic tool that could aid in management of cardiovascular disease and timing of surgical intervention. Furthermore, 4D flow CMR gives the opportunity to further unravel the influence of different surgical reconstruction methods on the cardiac and vascular function. However, longitudinal follow-up studies are needed to clarify the clinical value of 4D flow CMR-derived hemodynamic factors for risk stratification. Other future applications include the use of 4D flow CMR in the assessment of blood flow patterns in coronary arteries, which is currently still too challenging because of demands regarding the high spatial resolution needed for such small vessels and stringent necessity of cardiac motion correction. However, this application might become feasible when further improvements in hardware and imaging at high field strength become available.
